# The mediating role of emotional empathy in the relationship between adverse childhood experiences and misophonia: a structural equation modeling

**DOI:** 10.3389/fpsyg.2026.1771797

**Published:** 2026-05-11

**Authors:** Sevgi Koroglu Gokbel, Gulgun Durat

**Affiliations:** 1 Department of Nursing, Faculty of Health Sciences, Sakarya University, Serdivan, Sakarya, Türkiye; 2 Institute of Health Sciences, Sakarya University, Serdivan, Sakarya, Türkiye

**Keywords:** adverse childhood experiences, emotional intelligence, empathy, misophonia, violence

## Abstract

**Background:**

Misophonia is characterized by intense anger, disgust, and physiological arousal in response to specific everyday sounds. Recent studies suggest that this condition may be linked not only to sensory sensitivity but also to emotional processing and developmental experiences. Adverse childhood experiences have been found to be associated with lasting effects on emotional regulation, stress response, and empathic sensitivity. The aim of this study is to examine the mediating role of emotional empathy in the relationship between adverse childhood experiences and misophonia.

**Methods:**

This cross-sectional online study was conducted in Türkiye between September and November 2025, and 369 adults who met the diagnostic criteria for misophonia were included in the study. Data were collected using the Adverse Childhood Experiences (ACE) Scale, the Cognitive and Emotional Empathy Scale (emotional empathy subscale), and the Misophonia Scale. Direct and indirect relationships between variables were analyzed using structural equation modeling (SEM), and indirect effects were tested using a bootstrap method with 5,000 resamples.

**Results:**

The analysis results showed that adverse childhood experiences were positively associated with emotional empathy (*β* = 0.272, *p* = 0.001). In addition, emotional empathy levels were found to be significantly associated with the severity of misophonia. Mediation analysis indicated that emotional empathy played a partial mediating role in the relationship between ACE and misophonia. The indirect effect accounted for approximately 25% of the total effect. The structural model demonstrated a good level of fit (χ^2^/df = 1.588, RMSEA = 0.040, SRMR = 0.051, CFI = 0.945).

**Conclusion:**

The findings of this cross-sectional study suggest that developmental and emotional processes may be associated with misophonia. Emotional sensitivity associated with adverse experiences during childhood, alongside emotional empathy, may be linked to misophonic reactions. These findings suggest that focusing solely on trigger sounds in treatment may be limited; approaches targeting trauma processing, emotion regulation and empathic hypersensitivity may be beneficial.

## 1 Introduction

Misophonia is a disorder characterized by intense emotional and physiological reactions to specific sounds known as ‘triggers’, such as chewing, smacking and breathing (Ferrer-Torres and Giménez-Llort, 2022). The term “misophonia” was first defined in 2001 by Jastreboff and Jastreboff as a condition in which individuals exhibit abnormal reactions to specific sounds and frequently experience significant distress or anger (Jastreboff and Jastreboff, 2001). Since then, this condition has been addressed within audiological contexts and viewed as a symptom that has been under-reported within the broader context of sensory processing disorders (Duddy and Oeding, 2014). However, as research has expanded, its psychological dimension—including its association with comorbid psychiatric disorders—has begun to be investigated. Today, research into the psychological nature of misophonia is increasing (Jager et al., 2020; Rouw and Erfanian, 2018). In misophonia, triggers are predominantly repetitive everyday sounds such as chewing, smacking, pen clicking and breathing (Enzler et al., 2021). These sounds elicit deep emotional responses in individuals, such as anger, anxiety and disgust (Sweedo et al., 2022). These reactions are frequently accompanied by autonomic nervous system responses such as increased heart rate, galvanic skin response, and sweating (Sweedo et al., 2022). Individuals develop various strategies to avoid disturbing sounds or situations (Enzler et al., 2021; Simner et al., 2024). This phenomenon can lead some people to avoid social settings or to cover their ears to protect themselves from triggering sounds (Enzler et al., 2021). However, these avoidance behaviors can, over time, increase feelings of distress and loneliness and lead to social isolation (Simner et al., 2024). Symptoms of misophonia negatively impact school, social and professional life, as well as family relationships (Guzick et al., 2024; Schröder et al., 2013) and are associated with a decline in daily functioning and quality of life (Schröder et al., 2013). Current findings suggest that misophonia cannot be explained by auditory sensitivity alone; past experiences, contextual evaluations and emotional processing also play significant roles (Berger et al., 2024; Cerliani and Rouw, 2020; Savard et al., 2022; Wang et al., 2022).

Adverse childhood experiences (ACEs) are stressful and traumatic experiences occurring during childhood that have a negative impact on an individual’s mental, emotional and physical health. These include categories such as physical, emotional and sexual abuse, as well as neglect, mental illness within the family, substance abuse, domestic violence, and parental separation or imprisonment (Felitti et al., 1998; Martins et al., 2025). ACEs have been found to be associated with lasting effects on individuals’ biological and psychological functioning. These experiences have been linked to changes in the hypothalamic–pituitary–adrenal (HPA) axis, and are associated with increased cortisol levels and heightened stress sensitivity (Murphy et al., 2022). These changes in the HPA axis may be linked to individuals exhibiting higher stress responses to everyday challenges (Murphy et al., 2022). It is also associated with mental disorders such as depression, anxiety and post-traumatic stress disorder (PTSD) (Shalaby, 2024; Zhang et al., 2025). Attachment styles may be associated with difficulties in individuals’ emotional regulation skills. Adverse early-life experiences are linked to difficulties in the processes of understanding, expressing and controlling emotions, and may impair the capacity to respond adaptively to stress (Fontanil et al., 2021). Characterized by disturbances in attachment patterns, this process sees the misinterpretation of emotional cues and interpersonal conflicts becoming increasingly pronounced (Ashrafi et al., 2021). The cumulative effect of traumatic experiences may be associated with a decline in the ability to assess social cues and resolve conflicts; chronic stress and cognitive-emotional fluctuations have been found to be linked to a tendency towards maladaptive coping behaviors such as substance use and self-harm (Lansing et al., 2023). These psychobiological processes provide a framework that may explain the possible link between ACE and misophonia. Misophonia typically emerges during childhood, and individuals develop intense emotional reactions to specific sounds during this period (Bodo et al., 2024). Stress system dysregulation associated with ACEs may be linked to increased sensitivity to auditory stimuli (Kuzminskaite et al., 2020). The associated emotional dysregulation may be linked to individuals exhibiting more intense and difficult-to-control emotional responses to everyday sounds that trigger misophonia (Hartling et al., 2019) Chronic stress and difficulties with impulse control observed in individuals with a history of ACE have been found to be associated with the anger, disgust and avoidance behaviors reported in misophonia (Iob et al., 2021). The increased physiological arousal, elevated heart rate and anger responses observed in misophonia may be linked to sensitized stress response systems associated with past traumatic experiences (Leroux et al., 2023). Persistent changes in the HPA axis have been linked to heightened physiological and emotional responses to harmless sounds and may reinforce the sensitivity-distress cycle (Kuzminskaite et al., 2020; Iob et al., 2021). Therefore, misophonia can be considered a phenomenon associated with childhood experiences shaped via the stress system (Bodo et al., 2024; Hartling et al., 2019; Leroux et al., 2023).

It is thought that ACE’s are associated with an individual’s emotional processing patterns and may be linked to excessive emotional responses in misophonia (McGeoch and Rouw, 2020). In this context, it has been reported that individuals with misophonia tend to have a predisposition towards emotional empathy, defined as the tendency to perceive and share others’ emotions (Uribe et al., 2019). These findings suggest that emotional empathy may play a mediating role in the relationship between ACE and misophonia. Emotional empathy is a complex neurophysiological process supported by the limbic and paralimbic systems responsible for processing emotions (Pasquini et al., 2020). In particular, the anterior insula and anterior cingulate cortex (ACC) play a critical role in experiencing and expressing empathy (Penagos-Corzo et al., 2022). Furthermore, the connection between the orbitofrontal cortex and the limbic regions via the right uncinate fasciculus has been found to be associated with the integration of emotional information and the regulation of social responses; damage to this connection has been linked to a reduction in empathy (Girn et al., 2024). The mirror neuron system is a network that triggers similar neural patterns in an individual’s own brain whilst observing others’ behaviors or emotions (Penagos-Corzo et al., 2022). This system is regarded as the neurophysiological basis of empathic reflection and is associated with the capacity to share others’ emotions (Zhou et al., 2020). Functional neuroimaging studies have shown that when individuals witness others’ emotions, brain regions that are activated when they experience their own emotions are similarly activated (Uribe et al., 2019; Penagos-Corzo et al., 2022). These neurological mechanisms provide a framework that may explain the possible link between emotional empathy and misophonia (McGeoch and Rouw, 2020). Indeed, it has been reported that there is increased activity in the anterior insula in individuals with misophonia, and that these individuals process trigger sounds more intensely (Cerliani and Rouw, 2020; Hansen et al., 2022). Furthermore, it is stated that distinct connectivity patterns between the anterior insula and other brain regions are observed when these individuals are exposed to trigger sounds. The increased activity in the anterior insula has been found to be associated with emotional and autonomic responses and is considered one of the possible neurobiological underpinnings of the intense emotional responses observed in misophonia (Kumar et al., 2021). Furthermore, it is noted that in response to trigger sounds associated with orofacial movements in misophonia, distinct activation patterns have been reported in regions linked to the mirror neuron system, such as the ventral premotor cortex (Kumar et al., 2021). When these findings are considered together, it can be suggested that ACE may be associated with an individual’s capacity for emotional empathy, and that this association may be linked to the emotional responses observed in misophonia.

In this context, the primary aim of this study is to investigate whether emotional empathy plays a mediating role in the relationship between negative childhood experiences and symptoms of misophonia, using structural equation modeling. This study aims to understand the role of early-life adverse experiences and emotional empathy processes in the development of misophonia, and to examine how these processes might be addressed in therapeutic interventions.

In this context, the hypotheses of the study are as follows:

*H1*: The level of misophonia increases as adverse childhood experiences increase.

*H2*: The level of emotional empathy increases as adverse childhood experiences increase.

*H3*: As emotional empathy levels increase, misophonia levels increase.

*H4*: Emotional empathy plays a mediating role in the relationship between childhood negative experiences and misophonia.

## 2 Method

### 2.1 Setting and sample

This descriptive cross-sectional study was conducted online in Turkey between September and November 2025. Participants were selected via online platforms such as WhatsApp and Instagram using purposive sampling. Inclusion criteria for interested participants were (1) reporting at least three triggering sounds, (2) meeting all diagnostic criteria in the questionnaire based on the misophonia diagnostic criteria proposed by Schröder et al. (2013), and (3) being 18 years of age or older. The diagnostic criteria for misophonia proposed by Schröder et al. (2013) are as follows:

1. Feeling intense irritation, disgust, or anger toward specific human-generated sounds (e.g., chewing, breathing, clicking sounds),
2. These reactions reach a level that challenges the person’s self-control or causes fear of losing control,
3. Being aware that the reactions are excessive or unreasonable,
4. Avoiding triggering stimuli or experiencing significant distress when exposed to them,
5. These reactions negatively impact social, academic, or occupational functioning.

Exclusion criteria for interested participants: (1) having a neurological disease within the last year, (2) having a diagnosis of psychiatric disorder as the primary diagnosis, and (3) having tinnitus or hyperacusis.

Data were collected from 369 participants in accordance with the inclusion and exclusion criteria for the study, and the analyses were carried out on this sample. Sample adequacy specific to structural equation modelling was assessed using a *post hoc* power analysis based on the RMSEA. Based on the model’s degrees of freedom (df = 116), sample size (N = 369), *α* = 0.05, an RMSEA of 0.05 for good fit, and an RMSEA of 0.08 as the comparison value, the analysis determined that the statistical power was 0.999. This finding indicates that the current sample size exceeds the recommended level and is sufficient for testing the structural equation model.

### 2.2 Recruitment and data collection

An announcement informing participants about the purpose and scope of the study was shared on online platforms. This announcement included the researcher’s contact information. Volunteers wishing to participate in the study via the announcement were directed to a pre-screening form based on the criteria developed by Schröder et al. (2013). This form was used to assess participants’ eligibility for inclusion. Participants who did not meet the exclusion and inclusion criteria were automatically excluded from the study and could not proceed to the next steps.

Participants who met the eligibility criteria were informed by the system about the purpose and scope of the study, data privacy principles, potential benefits, and their right to withdraw from the study at any time; they were then asked to complete an online informed consent form. Participants who gave their consent completed the questionnaire and measurement tools online via the same link. All questions were mandatory and could only be completed once. The data collection process took approximately 3 months and was completed in November 2025.

### 2.3 Data collection tools

Data on participants’ demographic characteristics (gender, age, status, education level, and income level) were collected using a Personal Information Form developed by the researchers.

The Adverse Childhood Experiences (ACE) Scale was developed in 1997 by the Centers for Disease Control and Prevention (CDC) and Permanente. It is a self-report measurement tool designed to identify adverse experiences individuals were exposed to between the ages of 0 and 18 (Felitti et al., 1998). The Turkish validity and reliability study was conducted by Gündüz et al. (2018). The scale consists of 10 items, and responses are either “Yes” or “No.” The scale includes traumatic life events such as physical abuse, emotional neglect, sexual abuse, domestic violence, parental separation, and parental substance use. The score obtained from the scale ranges from 0 to 10. A high total score indicates that the individual was exposed to more negative experiences during childhood and may carry potential psychological risks (Gündüz et al., 2018). In this study, the Cronbach’s alpha coefficient of the scale is 0.72. In the structural equation modeling, the ACE variable was treated as a latent construct, and the items of the scale were included in the model as direct indicator variables.

The Cognitive and Emotional Empathy Scale was developed by Reniers et al. (2011). It is a self-report scale that assesses individuals’ levels of understanding others’ feelings and thoughts (cognitive empathy) and sharing these feelings emotionally (emotional empathy). The Turkish validity and reliability study of the 31-item scale was conducted by Gıca et al. (2021). The scale assesses individuals’ empathy levels under two main dimensions: (1) cognitive empathy, (2) emotional empathy. The emotional empathy main dimension consists of five subdimensions: emotion contagion, proximal responsivity, and peripheral responsivity; the cognitive empathy main dimension consists of two subdimensions: perspective- taking and online simulation. The scale items are rated on a 4-point Likert scale with response options of “strongly agree,” “agree,” “disagree,” and “strongly disagree” (Gıca et al., 2021). In this study, the Cronbach’s alpha coefficient for the scale’s emotional empathy main dimension is 0.71. In the structural equation modeling, the emotional empathy variable was included in the model as a latent construct, with its subdimensions used as indicator variables.

The Misophonia Scale was developed by Koroglu and Durat (2024) to assess the level of misophonia. The scale is a self-report measurement tool that assesses the frequency and severity of individuals’ misophonia-related symptoms. The 30-item scale is structured using a 5-point Likert-type rating system (1 = Strongly agree – 5 = Strongly disagree). The total score that can be obtained ranges from 30 to 150. As the score obtained from the scale decreases, the individual’s level of misophonia is considered to increase. The scale consists of four subscales: triggers and responses, self-control, coping strategies, and quality of life. A cut-off point of 60.5 points has been determined for the scale, and it is suggested that individuals scoring below this value may need to consult a psychiatrist (Koroglu and Durat, 2024). The Cronbach’s alpha coefficient for the scale in this study is 0.93. As lower scores on this scale indicate a higher severity of misophonia, this inverse relationship has been taken into account when interpreting the coefficients obtained in the analyses. Accordingly, negative correlations correspond to an increase in the severity of misophonia. In the structural equation modeling, the misophonia variable was included in the model as a latent construct, with the subdimensions of the scale used as indicator variables.

### 2.4 Data analysis

Data were analyzed using IBM SPSS Statistics for Windows Version 25.0 and R software Version 4.4.2. Structural equation modeling (SEM) was performed in the R environment using the lavaan package. Descriptive statistics are presented as mean and standard deviation or median (minimum–maximum) for continuous variables and as frequency and percentage for categorical variables.

The normality assumption was evaluated using graphical methods (Q–Q plots and histograms) as well as skewness and kurtosis values. Graphical inspections indicated that the distributions were approximately normal. The skewness and kurtosis values were 0.393 and −0.805 for the ACE variable, −0.039 and −0.583 for the emotional empathy variable, and −0.017 and −0.767 for the misophonia variable, respectively. These values were found to be within acceptable limits (|skewness| < 2 and |kurtosis| < 7) (Kline, 2011). Therefore, Pearson correlation analysis was used to examine the relationships between the variables. Since the variables used in this study were based on total or subscale scores obtained from the scales, they were treated as continuous variables. Additionally, because the normality assumption was satisfied and the study aimed to test a theoretically specified model, a covariance-based structural equation modeling approach was preferred.

Structural equation modeling was used in the study to test the direct and indirect effects of conscious awareness on misophonia in individuals with misophonia. The model results obtained were visualized with a path diagram. The level of model fit was assessed using multiple fit indices widely accepted in the literature. A chi-square/degrees of freedom ratio (χ^2^/df) value of ≤ 2 indicates excellent fit, 2–3 indicates good fit, 3–5 indicates acceptable fit, and > 5 indicates poor fit (Kline, 2011). RMSEA (Root Mean Square Error of Approximation) and SRMR (Standardized Root Mean Square Residual) values below 0.08 are considered a good fit (Hu and Bentler, 1999). For the comparative fit indices CFI (Comparative Fit Index) and TLI (Tucker-Lewis Index), values of 0.90 and above indicate acceptable fit; for NFI (Normed Fit Index), values close to or above 0.90 indicate adequate fit (Bentler, 1990; Hu and Bentler, 1999; Bentler and Bonett, 1980). Furthermore, GFI (Goodness of Fit Index) and AGFI (Adjusted Goodness of Fit Index) values above 0.90 indicate that the model fits the data well (Jöreskog and Sörbom, 1993).

The convergent validity and composite reliability of the measurement model were evaluated using average variance extracted (AVE) and composite reliability (CR) values, while discriminant validity was assessed using the Fornell–Larcker criterion.

Furthermore, the 5,000-sample bootstrap method was applied to obtain more reliable standard errors and confidence intervals for indirect and total effects. Findings are reported with confidence intervals; the level of statistical significance was set at *p* < 0.05.

### 2.5 Ethical considerations

Ethical approval for this study was obtained from the Ethics Committee of the Faculty of Social and Human Sciences at Sakarya University (No: E-61923333-050.99-555154). Consent was obtained from all participants via an “Informed Consent Form” through an online platform. All procedures were conducted in accordance with the ethical standards of institutional and national research committees and the principles of the 1964 Helsinki Declaration, as revised in 2000.

## 3 Results

### 3.1 General characteristics

A total of 369 individuals participated in the study. The average age of participants was 26.57 ± 5.43, ranging from 20 to 55 years old. 72.1% (*n* = 266) of the participants were female, and 27.9% (*n* = 103) were male; 76.4% (*n* = 282) were single, and 23.6% (*n* = 87) were married. Furthermore, 23.6% (*n* = 87) of participants had a high school education, 14.1% (*n* = 52) had an associate’s degree, 35.5% (*n* = 131) had a bachelor’s degree, and 26.8% (*n* = 99) had a postgraduate degree. In terms of income, 34.2% of participants (*n* = 126) had an income less than their expenses, 39.8% (*n* = 147) had an income equal to their expenses, and 26.0% (*n* = 96) had an income greater than their expenses. The demographic characteristics of the participants are presented in Table 1.

**Table 1:** Demographic characteristics of the participants (*n* = 369).

Variable	Statistics
Age, mean ± standard deviation (min–max)	26.57 ± 5.43 (20–55)
Gender, *n* (%)	
Female	266 (72.1%)
Male	103 (27.9%)
Marital status, *n* (%)	
Married	87 (23.6%)
Single	282 (76.4%)
Educational status, *n* (%)	
High school	87 (23.6%)
Associate’s degree	52 (14.1%)
Bachelor’s degree	131 (35.5%)
Postgraduate degree	99 (26.8%)
Income status, *n* (%)	
Income less than expenses	126 (34.2%)
Income equal to expenses	147 (39.8%)
Income greater than expenses	96 (26.0%)

### 3.2 Correlation analysis

The Pearson correlation analysis between ACEs, emotional empathy, and misophonia among individuals with misophonia is shown in Table 2. ACEs showed a negative correlation with misophonia (*r* = −0.19, *p* < 0.001). As lower scores on the misophonia scale represent higher severity, this finding indicates a positive relationship between ACE level and the severity of misophonia. Similarly, a negative correlation was found between emotional empathy and misophonia (*r* = −0.26, *p* < 0.001). Given the reverse-scored nature of the scale, this result indicates a positive relationship between emotional empathy and the severity of misophonia (Table 2).

**Table 2:** Correlations among the variables (*n* = 369).

Variable		ACEs	Emotional Empathy				Misophonia				
		1	2	3	4	5	6	7	8	9	10
ACEs	1	1									
Emotional empathy	2	0.18**	1								
	3	0.18**	0.83**	1							
	4	0.21**	0.51**	0.51**	1						
	5	0.23**	0.89**	0.89**	0.79**	1					
Misophonia	6	−0.13*	−0.24**	−0.24**	−0.17**	−0.25**	1				
	7	−0.14**	−0.23**	−0.20**	−0.13*	−0.22**	0.52**	1			
	8	−0.13*	−0.18**	−0.17**	−0.07	−0.16**	0.49**	0.51**	1		
	9	−0.19**	−0.16**	−0.17**	−0.15**	−0.19**	0.39**	0.61**	0.43**	1	
	10	−0.19**	−0.25**	−0.25**	−0.17**	−0.26**	0.75**	0.80**	0.72**	0.84**	1

**p < 0.01, *p < 0.05. 1: The Adverse Childhood Experiences (ACE) Scale; 2: Emotion contagion, 3: Proximal responsivity, 4: Peripheral responsivity, 5: Emotional Empathy Scale; 6: Triggers and responses, 7: Self-control, 8: Coping strategies, 9: Quality of life, 10: Misophonia Scale.

### 3.3 Validation results of the research model

The fit indices indicated that the proposed structural equation model showed good fit to the data: χ^2^(116) = 184.265, *p* = 0.043, χ^2^/df = 1.588, RMSEA = 0.040, SRMR = 0.051, CFI = 0.945, TLI = 0.935, NFI = 0.866, GFI = 0.947, and AGFI = 0.930.

In addition, the convergent validity and composite reliability of the measurement model were evaluated using AVE and CR values. Emotional empathy (CR = 0.845, AVE = 0.655) and misophonia (CR = 0.798, AVE = 0.499) demonstrated acceptable reliability and convergent validity. The AVE and CR values of the ACE construct were relatively low (CR = 0.565, AVE = 0.122). This may be expected because the ACE scale represents a heterogeneous construct encompassing different types of traumatic experiences and its items are scored in a binary (0–1) format.

Discriminant validity was evaluated using the Fornell–Larcker criterion. The square root of the AVE values for each construct was greater than the correlations among the constructs, indicating that discriminant validity was established (Table 3).

**Table 3:** Discriminant validity of the constructs (Fornell–Larcker criterion).

Construct	ACEs	Emotional Empathy	Misophonia
ACEs	**0.349**		
Emotional Empathy	0.272	**0.809**	
Misophonia	−0.272	−0.305	**0.707**

Bold values represent the square root of the average variance extracted (√AVE) for each construct.

### 3.4 Path analysis

Path coefficient estimation and significance testing were performed to verify the relationships between variables using SEM. According to the analysis results, a significant and positive relationship was found between ACEs and emotional empathy (*β* = 0.272, *p* = 0.001). This finding indicates a positive relationship between ACE levels and emotional empathy (Table 4).

**Table 4:** Path analysis.

Path	Effect (unstandardized *β*)	SE	CR	*p*	Effect (standardized *β*)
ACEs ⟶ Emotional Empathy	1.967	0.591	3.330	0.001	0.272
ACEs ⟶ Misophonia	−4.413	2.134	−2.068	0.039	−0.204
Emotional Empathy ⟶ Misophonia	−0.749	0.207	−3.611	<0.001	−0.250

SE: Standard error; CR: Confidence interval; ACEs: Adverse childhood experiences.

The negative correlation between ACEs and misophonia (*r* = −0.19, *p* < 0.001), when the scale’s reverse-scored structure is taken into account, indicates a positive relationship between the two variables.

Similarly, the negative correlation between emotional empathy and misophonia (*r* = −0.26, *p* < 0.001) indicates a positive relationship when reverse scoring is taken into account.

### 3.5 Impact analysis

The Bootstrap method was used to test the mediating role of emotional empathy in the effect of ACEs on misophonia. The analyses revealed that the direct effect of ACEs on misophonia was *β* = −4.413, and this effect was statistically significant at the *p* = 0.039 level. Given the reverse-scored nature of the misophonia scale, this negative coefficient indicates a positive relationship between the level of ACEs and the severity of misophonia.

Furthermore, the effect of ACE on emotional empathy is positive and significant (*β* = 1.967, *p* = 0.001). Conversely, emotional empathy exerts a negative and significant effect on misophonia (*β* = −0.749, *p* < 0.001). Given the direction of the scale, this finding indicates a positive correlation between emotional empathy and the severity of misophonia.

The indirect effect calculated based on these relationships is *β* = −1.473, which is statistically significant at the *p* = 0.016 level. The fact that the 95% confidence interval falls within the range [−2.875, −0.485] and does not include zero indicates that the mediating effect is statistically reliable. Taking reverse scoring into account, this indirect effect suggests that the relationship between ACE exposure and the severity of misophonia may be associated with emotional empathy.

The total effect, obtained by combining direct and indirect effects, was calculated as *β* = −5.887, and this effect was found to be significant at the *p* = 0.008 level (95% CI [−10.942, −2.346]). Given the scale’s reverse-scored nature, the total effect indicates a general positive relationship between ACE level and the severity of misophonia.

**Figure 1:**
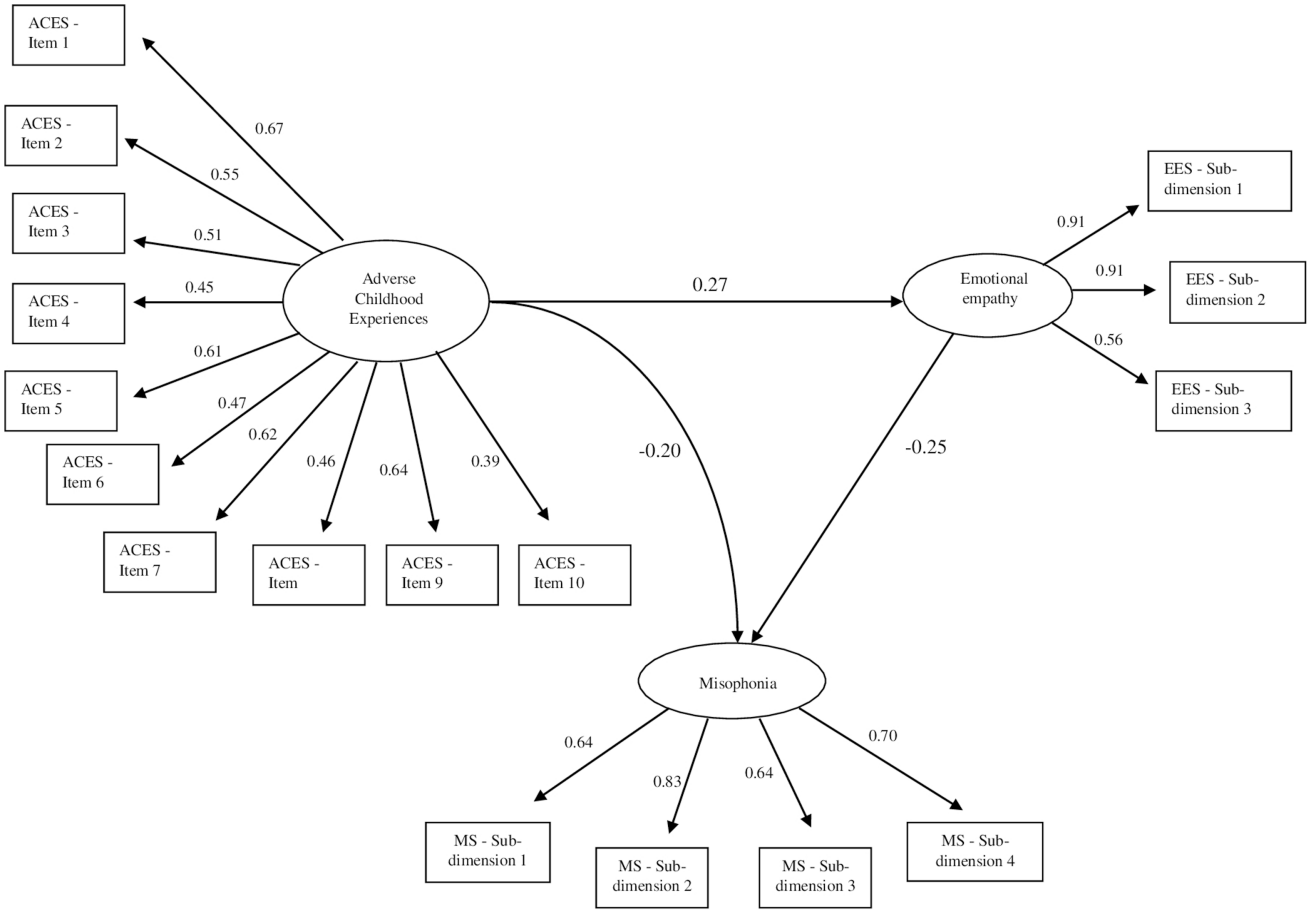
Structural equation model showing the relationships between adverse childhood experiences (ACEs), emotional empathy, and misophonia.

The ratio of the indirect effect to the total effect has been calculated as approximately 25%. These findings suggest that the level of emotional empathy plays a significant and partial mediating role in the relationship between ACE and misophonia. The relevant path coefficients and statistical values are presented in Table 5; the path relationships in the model are illustrated in Figure 1.

**Table 5:** Mediating effect of emotional empathy.

Path/effect	Effect coefficient (β)	Standard error (SE)	*p*	95% confidence interval
Direct effects				
ACEs ⟶ Emotional empathy	1.967	0.591	0.001	[0.901, 3.254]
ACEs ⟶ Misophonia	−4.413	2.134	0.039	[−9.237, −0.808]
Emotional empathy ⟶ Misophonia	−0.749	0.207	<0.001	[−1.152, −0.350]
Indirect effect				
ACEs ⟶ Emotional empathy ⟶ Misophonia	−1.473	0.611	0.016	[−2.875, −0.485]
Total effect				
ACEs ⟶ Misophonia	−5.887	2.208	0.008	[−10.942, −2.346]

ACEs: Adverse childhood experiences.

In addition, a multiple linear regression analysis was conducted to assess the potential effect of the gender variable, and gender was included in the model as a control variable. The results of the analysis showed that the direction and statistical significance of the relationships between emotional empathy and ACE’s and misophonia remained unchanged. Furthermore, no significant effect of gender on misophonia was found (*β* = −0.061, *p* = 0.223). These findings support the notion that the relationships observed are independent of gender distribution.

## 4 Discussion

Understanding the psychological mechanisms associated with misophonia in patients with the condition is important for grasping how these processes can be used in therapeutic interventions. In this context, this study examines the mediating role of emotional empathy in the relationship between adverse childhood experiences (ACEs) and symptoms of misophonia.

Due to the reverse-scored nature of the misophonia scale used in this study (where lower scores represent higher severity), the negative coefficients obtained in the analyses conceptually correspond to an increase in the level of misophonia. This factor was specifically taken into account when interpreting the findings, and all results were evaluated within this framework.

The findings reveal that ACE are significantly associated with the severity of misophonia both directly and indirectly via increased emotional empathy, and that all proposed hypotheses are supported.

The findings of this study indicate that higher ACE scores are associated with increased severity of misophonia. Findings in the literature support this conclusion. Guetta et al. (2024), in their study of adults with misophonia, found that the number of traumatic stressors and adverse life events did not directly predict the severity of misophonia. However, they demonstrated a significant association between more intense misophonic reactions and symptoms of hyperarousal associated with PTSD. In a study by Stalias-Mantzikos et al. (2023) on adults with misophonia, the link between early maladaptive schemas and misophonic distress and reactions was investigated. The study identified schemas of poor self-regulation, social isolation, and vulnerability as significant predictors of misophonic distress and reactions. Early maladaptive schemas stem from unmet emotional needs during childhood (Sójta and Strzelecki, 2023), suggesting that ACE plays a significant role in misophonia (Stalias-Mantzikos et al., 2023). Numerous studies highlight PTSD as a frequently reported comorbidity in patients with misophonia (Rouw and Erfanian, 2018; Smit et al., 2022). In this context, ACE, which may underlie the psychosocial and neurobiological basis of PTSD, is thought to be linked to misophonia. Based on these findings in the literature, the relationship between ACE and misophonia may manifest in various ways. Early-life experiences of neglect and abuse may be associated with hypersensitivity in the brain’s stress system, known as the HPA axis. Consequently, individuals may exhibit high physiological arousal in response to environmental stimuli. This biological sensitivity, when combined with processing changes in the limbic system, may be associated with the perception of even neutral or mild stimuli as threatening (Schalinski et al., 2019). Individuals with misophonia exhibit a response pattern characterized by anger, physical tension, and impulsive reactions to triggers (Guetta et al., 2024). Changes in the limbic system observed in individuals with a history of childhood trauma (Lu et al., 2024) are also observed in misophonia (Kumar et al., 2017). Another possibility is that ACEs are associated with difficulties in emotional regulation skills. In this case, individuals may struggle to regulate emotions such as anger and anxiety (Ion et al., 2023). The difficulties experienced by individuals with misophonia in controlling emotions such as anger, anxiety, and disgust (Guetta et al., 2022) and the development of misophonia symptoms during childhood support this view (Siepsiak et al., 2023).

The findings of this study indicate that emotional empathy plays a partial mediating role between ACE scores and the severity of misophonia. This result suggests that high ACE scores increase the severity of misophonia by enhancing emotional empathy. Although there are no studies in the literature that directly examine the mediating role of emotional empathy, studies have been conducted in various populations. Research indicates that adults who encountered a traumatic event in childhood exhibit, on average, elevated levels of empathy relative to those who did not. This difference was particularly pronounced in the components of emotional empathy (Greenberg et al., 2018). A study conducted on adults found that certain types of trauma (particularly sexual abuse) can increase emotional empathy, whereas others (emotional/physical abuse) may weaken emotional sensitivity or indirectly lead to impairment (Mueller et al., 2020). A study of sex offenders indicated that childhood emotional abuse enhances emotional empathy; however, this enhancement correlates with elevated psychopathology and emotional instability (Petruccelli et al., 2022). However, there are also findings in the literature that contradict this perspective. Irvin and Collin (2024) conducted a study with female participants and found that adverse childhood experiences (neglect, abuse, family dysfunction, etc.) do not have a direct effect on levels of emotional empathy in women. Emotional neglect and abuse experienced during childhood are more prevalent among individuals with eating disorders, and these individuals’ levels of emotional empathy are lower compared to the general population (Meneguzzo et al., 2025). Several factors explain this difference. Firstly, the type of trauma may affect empathy capacity in different ways. A history of emotional neglect may reduce empathy capacity due to insufficient activation in the brain’s social–emotional processing regions (Li et al., 2024), whereas empathy increases in individuals who have experienced sexual abuse (Benz et al., 2023). Secondly, supportive caregiving relationships and therapeutic interventions that mitigate the effects of childhood trauma may contribute to the development of empathic sensitivity (Ray et al., 2022; Van Doorn et al., 2024). Thirdly, the characteristics and gender of the working population may play a role. While empathy is often suppressed or dysfunctional in clinical samples, this effect is weaker or more balanced in general population samples (Cerqueira and Almeida, 2023). While women generally demonstrate greater empathy, men may be more prone to emotional withdrawal (dos Santos et al., 2024; Komatsu et al., 2024). The fact that our study did not address a specific type of neglect or abuse may have led to this result, given the predominance of women in the general population sample.

Furthermore, the emotional empathy concept assessed in this study requires careful interpretation. Empathy is a multidimensional construct associated with prosocial behavior, referring to the capacity to understand others’ emotions, adopt their perspective, and respond to these emotions in a regulated and other-oriented manner (Wu and Hong, 2022). In contrast, emotional contagion—which can be confused with emotional empathy—is a more primitive process characterized by an individual’s automatic, rapid, and uncontrolled response to others’ emotions and is frequently associated with personal distress and increased emotional load (Keysers et al., 2022). Indeed, it has been demonstrated that emotional contagion does not always align with empathic concern or prosocial motivation and in some cases operates independently of these processes (Blunden et al., 2024). Moreover, it has been posited that self-report measures of empathy may indicate an individual’s emotional sensitivity and personal distress rather than a regulated empathic capacity (Eisenberg et al., 2024; Plusquellec et al., 2025). In this context, it is thought that the construct assessed as ‘emotional empathy’ in the present study may reflect increased emotional sensitivity and reactivity rather than a prosocial empathic capacity. Such reactivity may be associated with the individual exhibiting faster and more intense emotional responses to environmental stimuli and increased sensitivity to such stimuli (Hara et al., 2025; Morellini et al., 2023). Consequently, the findings suggest that the relationship between increased levels of emotional empathy and the severity of misophonia may be explained through heightened emotional reactivity rather than the direct influence of empathic processes.

Theoretical and neurobiological explanations regarding how trauma may affect empathy also support our findings. It has been suggested that adverse childhood experiences may be associated with the development of hypersensitivity to environmental stimuli and threat-focused hypervigilance (Seitz et al., 2021; Dell’Acqua et al., 2025). This process may increase defense-based emotional empathy by creating heightened sensitivity to others’ emotional expressions and behaviors (Benz et al., 2023; Levy et al., 2019). Such heightened empathic reactivity may pave the way for interpreting certain interpersonal sounds as threatening or distressing stimuli (Levy et al., 2019; Seitz et al., 2021). Neuroimaging studies indicate that heightened responses associated with misophonia are linked to increased activation in brain regions related to empathy and emotional processing, particularly the anterior insula and amygdala (Kumar et al., 2017). Consequently, emotional empathy may be considered a potential mediating mechanism at both psychological and neurobiological levels in the relationship between traumatic childhood experiences and the severity of misophonia (Guetta et al., 2022). Consequently, focusing solely on sound triggers in misophonia intervention may prove limited. Increased emotional empathy reactivity associated with traumatic experiences suggests that approaches in treatment focusing on emotion regulation, trauma processing, and reducing excessive emotional resonance may be beneficial.

### 4.1 Limitations

This study has several limitations. The research was conducted in Turkey using online channels. The great importance placed on family privacy in Turkish culture and the principle of not discussing family matters outside the home’ may be associated with lower reporting rates of traumatic experiences, particularly those related to emotional neglect and emotional abuse (Yildiz et al., 2024). Culturally constrained emotional expression and ongoing authoritarian parenting practices may be linked to a reduced tendency for individuals to perceive childhood experiences as traumatic (Welkin et al., 2015). Cultural characteristics may increase social desirability and recall bias in self-report trauma assessments, and these factors may be associated with underreporting of trauma scores.

The scale measuring emotional empathy does not sufficiently distinguish between emotional sensitivity and emotional reactivity, which leaves it unclear whether empathy scores accurately represent the ability to genuinely share another person’s emotions or merely reflect reactivity stemming from increased emotional sensitivity and vulnerability following trauma. Recent research suggests that characteristics such as “being easily affected by others’ distress” may be confused with emotional reactivity in assessments of emotional empathy (Grevenstein, 2020; Lüönd et al., 2023). Furthermore, research indicates that women exhibit higher levels of empathy than men (Sommerlad et al., 2021) and that empathy tends to decrease with age (Grühn et al., 2008). The predominance of young people and women in the sample may be associated with higher levels of empathy and may have influenced relationships due to this demographic composition.

Thirdly, as misophonia is not currently recognized as an official diagnostic category in the DSM-5 and ICD-11, assessment methods rely on self-reports. Although participants were screened against the diagnostic criteria established by Schröder et al. (2013), no clinical validation was conducted; this is linked to the limited certainty of the diagnosis. In addition, participants in this study were selected through purposive sampling via online platforms. Since misophonia is not currently recognized as an official diagnostic category in the DSM-5 or ICD-11, a medical diagnostic criterion could not be used for participant inclusion. Therefore, sample selection was based on a self-report screening process, and the inability to establish a clinically verified patient group should be considered one of the important limitations of this study.

The cross-sectional design of the study prevents the establishment of causal relationships between variables and requires that the findings be interpreted only at a correlational level. Moreover, collecting the sample through online platforms may have excluded individuals with limited internet access. In addition, the predominance of young adults and women in the sample may have reduced the representativeness of the study and limited the generalizability of the findings.

### 4.2 Clinical and scientific implications

Future research should employ longitudinal designs to shed light on the causal dimensions of the interactions between ACE, emotional empathy, and misophonia. To determine whether emotional empathy truly functions as a mediating mechanism, it may be important to conduct studies that experimentally manipulate the empathic load.

Intervention studies evaluating the effect of methods such as Eye Movement Desensitization and Reprocessing (EMDR), schema therapy, or Acceptance and Commitment Therapy (ACT)—which focus on trauma and early schemas—on misophonia symptoms will enhance the clinical validity of the findings. Furthermore, empirically tested protocols focusing on cognitive restructuring, emotion regulation, and attention processes could enhance the empirical validity of cognitive-behavioral theories of misophonia. The development of multidisciplinary models that integrate psychological variables with neurobiological measures—such as functional magnetic resonance imaging (fMRI), electroencephalography (EEG), and autonomic nervous system indicators—may be useful for a comprehensive understanding of psychological mechanisms. Consequently, future research that examines various types of empathy and the subdimensions of childhood adversity in detail may facilitate a more nuanced and differentiated understanding of the processes influencing misophonia.

## 5 Conclusion

This study suggests that emotional empathy may play a mediating role in the link between ACE and misophonia. The results indicate that negative childhood experiences correlate with emotional processing and heightened sensitivity to misophonic stimuli. The study shows that emotional vulnerability, rather than mere sensory issues, may complexly link to misophonia. This comprehensive perspective offers a more detailed understanding of how emotional sensitivity developing in early life may be associated with the severity of misophonia symptoms.

The findings suggest that underlying processes may be linked to trauma-related emotional patterns and heightened empathic sensitivity; therefore, treatment approaches focusing solely on external auditory stimuli may be limited. Consequently, therapeutic treatments should focus on enhancing emotional awareness, regulating empathic over-activation, and addressing the unresolved emotional residues of childhood trauma. Interventions addressing early-stage schemas, trauma-related responses, and emotional reactivity may yield changes associated with the severity of misophonic reactions. These findings suggest that integrating emotional and developmental elements into a more holistic therapeutic approach may be important.

## Data Availability

The raw data supporting the conclusions of this article will be made available by the corresponding author upon reasonable request.

## Glossary

ACE: Adverse childhood experience
PTSD: Post-traumatic stress disorder
ACC: Anterior cingulate cortex
CDC: Centers for Disease Control and Prevention
SEM: Structural equation modeling
RMSEA: Root Mean Square Error of Approximation
SRMR: Standardized Root Mean Square Residual
CFI: Comparative Fit Index
TLI: Tucker-Lewis Index
NFI: Normed Fit Index
GFI: Goodness of Fit Index
AGFI: Adjusted Goodness of Fit Index
AVE: Average Variance Extracted
CR: Composite Reliability
HPA: Hypothalamic–pituitary–adrenal
DSM: Diagnostic and Statistical Manual
ICD: International Classification of Diseases
EMDR: Eye movement desensitization and reprocessing therapy
ACT: Acceptance and commitment therapy
fMRI: Functional magnetic resonance imaging
EEG: Electroencephalography
